# *Arylsulfatase I* is a prognostic biomarker for head and neck squamous cell carcinoma and Pan‐cancer

**DOI:** 10.1002/jcla.24600

**Published:** 2022-07-23

**Authors:** Yiming Shen, Zhengyu Wei, Chongchang Zhou, Jiangping Song, Jianing Wang, Jiada Wang, Linrong Wu, Shenzhe Fang, Zhisen Shen

**Affiliations:** ^1^ Department of Otolaryngology, Head and Neck Surgery, The Affiliated Lihuili Hospital Ningbo University Ningbo China; ^2^ Department of Otolaryngology, Head and Neck Surgery Ningbo Medical Center Lihuili Hospital Ningbo China; ^3^ Medical School of Ningbo University Ningbo China

**Keywords:** *ARSI*, drug sensitivity, GSEA, GSVA, HNSC, prognosis, tumor microenvironment

## Abstract

**Background:**

Sulfatase gene family members mediate various biological functions in tumor stroma and tumor cell environments. However, the expressions and prognostic value of *Arylsulfatase I* (*ARSI*), a sulfatase gene family member, in head and neck squamous cell carcinoma (HNSC) have not been fully established.

**Methods:**

Arylsulfatase I expressions in pan‐cancer were profiled using publicly available databases. Then, univariate Cox regression, Kaplan–Meier, and the Pearson's correlation analyses were performed to determine correlations between *ARSI* expressions and cancer prognosis, immune cell status, and drug sensitivity. Gene set variation analysis (GSVA) and gene set enrichment analysis (GSEA) were used to assess the potential mechanisms underlying *ARSI* functions in HNSC.

**Results:**

Arylsulfatase I was highly expressed in 15 cancer types, with significant expressions in HNSC. Elevated *ARSI* levels were associated with worse prognostic outcomes in HNSC patients. In addition, GSVA and GSEA showed that *ARSI* was highly involved in tumor cell escape and inflammatory responses. Expressions of *ARSI* negatively correlated with tumor mutation burden or microsatellite instability and positively correlated with immune‐related genes. Elevated *ARSI* expressions conferred poor tolerance to daporinad and sinularin, but increased cell sensitivity to dasatinib and XAV939.

**Conclusion:**

Arylsulfatase I is a promising prognostic and therapeutic target for HNSC.

## Graphical abstract

The work flow of the study. TCGA; The Cancer Genome Atlas, GTEx; Genotype‐Tissue Expression, GSVA; Gene Set Variation Analysis, GSEA, Gene Set Enrichment Analysis, *ARSI*; *Arylsulfatase I*, MSI; Microsatellite instability, TMB; Tumor mutational burden.

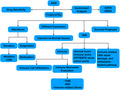

## 1 INTRODUCTION

Squamous cell carcinoma (SCC), which arises from precancerous lesions with atypical squamous proliferation, is one of the most common types of pathology in head and neck cancer.^1^ The head and neck squamous carcinoma (HNSC) often metastasizes to lymph nodes.^1^ Risk factors for HNSC include human papillomavirus (HPV) infections, tobacco consumption, and alcohol use.^2^ More than 500,000 patients with HNSC undergo radiotherapy and other therapeutic treatments annually. However, patients with head and neck cancer, especially men, have high recurrence and mortality rates.^3^ The lack of reliable, independent biomarkers for early diagnosis as well as prediction of survival and responses to treatment are a challenge to clinical management HNSC.

Chronic inflammation, immune escape, metabolic reprogramming, cellular senescence, and genome mutations are some of the mechanisms involved in carcinogenesis. However, these mechanisms have not been fully investigated in HNSC.^4^, ^5^, ^6^

*Arylsulfatase I* (*ARSI*) is one of the seventeen members of sulfatase gene family, whose aberrant expressions contribute to cancer cell migration.^7^, ^8^ *ARSI* is mainly expressed in embryonic tissues and is associated with tissue remodeling.^9^ However, the role of *ARSI* in HNSC is yet to be defined. We evaluated the genetic background of HNSC to characterize the significance of *ARSI* in HNSC progression. Transcriptome and clinical data were extracted from the Cancer Genome Atlas head and neck squamous carcinoma cohort (TCGA‐HNSC). Then, expressions of *ARSI* in all cancer types were evaluated, and its prognostic value, including in overall survival (OS), disease‐specific survival (DSS), disease‐free interval (DFI), and progression‐free interval (PFI), determined. The associations between *ARSI* and immune scores, stromal scores, ESTIMATE scores, and tumor purity were also evaluated. Besides, comprehensive analyses of the *ARSI* gene at tumor mutation burden (TMB) and microsatellite instability (MSI) levels were performed. Then, correlations between differential expressions of *ARSI* and anticancer drug sensitivity were assessed. Our findings highlight potential tumor immunotherapy targets and provide novel insights into precise diagnosis and early interventions to improve the survival rate of HNSC patients.

## 2 METHODS

### 2.1 Data collection and processing

The UCSC XENA website (https://xenabrowser.net/datapages/), which includes various transcriptomic datasets, such as the TCGA portal; the Genotype‐Tissue Expression (GETx) project and Cancer Cell Line Encyclopedia (CCLE) was used in this study. The data are publicly available and open‐ended, and require no ethics approval. We retrieved somatic cell mutations, CNAs, and methylation data on *ARSI* from tissue samples in 33 cancer types using cBioPortal. Genetic data types, including somatic cell mutations, DNA copy number alterations (CNAs), and DNA methylation, were integrated by cBioPortal (https://www.cbioportal.org/). One HNSC dataset (GSE41613) was downloaded from the Gene Expression Omnibus (GEO) database to validate the prognostic role of *ARSI* and the relationship between *ARSI* expressions and infiltrating immune cells.

### 2.2 Analysis of the relationship between *ARSI* and prognosis

The four major prognostic factors (OS, DSS, DFI, and PFI) were used to define the relationship between *ARSI* expressions and prognostic outcomes for 33 cancer types using univariate proportional hazards regression. Thereafter, Kaplan–Meier estimates and log‐rank tests were used to assess survival outcomes for several cancer types with elevated *ARSI* levels and poor prognosis (*p* < 0.05). The R packages “survival” (http://cran.rproject.org/web/packages/survival/index.html) and “survminer” (https://cran.r‐project.org/web/packages/survminer/index.html) were used in these analyses.

### 2.3 GSVA and GSEA

Using “GSVA” in R, Gene Set Variation Analysis (GSVA), a non‐parametric and unsupervised software algorithm, was employed to analyze the associations between *ARSI* expressions and hallmark gene sets, which represent well‐defined biological processes in cancers. In addition, gene set enrichment analysis (GSEA; http://www.broadinstitute.org/gsea/) was performed to investigate the significance of *ARSI* gene signatures. GSEA is a tool for analyzing genome microarray data, creating a molecular signature database based on known positions, characteristics, and functions of different gene sets.

### 2.4 Correlations between *ARSI* expressions and tumor microenvironment

We systematically analyzed the immune, stromal, and ESTIMATE scores as well as tumor purity in different cancer types using the “estimate” R package. Then, correlations between *ARSI* expressions and immune infiltrating scores of twenty‐four immune cells, which had been obtained from the Tumor Immune Evaluation Resource (TIMER) database, were evaluated. In addition, Pearson correlation analysis was performed to assess the relationship between *ARSI* expressions and infiltrating immune cells, including B cells, CD4+ T cells, CD8+ T cells, NK cells, mast cells, macrophages, dendritic cells, and neutrophils.

### 2.5 Correlations between *ARSI* expressions and TMB, MSI, Immune‐related genes (IRGs), or drug sensitivity

TMB, MSI, and IRGs are significant biomarkers in the tumor microenvironment (TME). The R software was used to assess the relationships between *ARSI* expressions and levels of TMB, MSI, or IRGs. Associations between *ARSI* gene expressions and small molecule drugs from Genomics of Drug Sensitivity in Cancer (GDSC, https://www.cancerrxgene.org) were also evaluated.

### 2.6 Statistical analysis

All statistical analyses were performed in R software (version 4.1.1). Differences in *ARSI* expressions were evaluated using the Student's *t*‐test or paired *t*‐test. Spearman correlation analyses were performed to establish correlations between *ARSI* expressions and drug IC50. *p < 0.05, **p < 0.01, ***p < 0.001, ****p < 0.0001, ns: non‐significant.

## 3 RESULTS

### 3.1 *ARSI* expressions in human cancers and normal tissues

**FIGURE 1:**
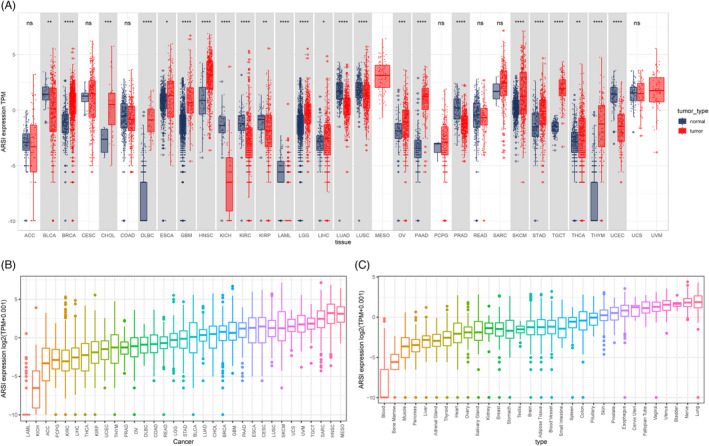
Differential expression of *ARSI*. (A) Pan cancer expression profile of *ARSI* from the Cancer Genome Atlas (TCGA) cohorts and GETx database. (B) *ARSI* expression in various tumor tissues based on TCGA. (C) *ARSI* expression in various normal tissues based on GETx. *p < 0.05, **p < 0.01, ***p < 0.001. ACC, adrenocortical carcinoma; BLCA, bladder urothelial carcinoma; BRCA, breast invasive carcinoma; CESC, cervical and endocervical cancers; CHOL, cholangiocarcinoma; COAD, colon adenocarcinoma; DLBC, lymphoid neoplasm diffuse large B‐cell lymphoma; ESCA, esophageal carcinoma; GBM, glioblastoma multiforme; HNSC, head and neck squamous cell carcinoma; KICH, kidney chromophobe; KIRC, kidney renal clear cell carcinoma; KIRP, kidney renal papillary cell carcinoma; LAML, acute myeloid leukemia; LGG, brain lower grade glioma; LIHC, liver hepatocellular carcinoma; LUAD, lung adenocarcinoma; LUSC, lung squamous cell carcinoma; MESO, mesothelioma; OV, ovarian serous cystadenocarcinoma; PAAD, pancreatic adenocarcinoma; PCPG, pheochromocytoma and paraganglioma; PRAD, prostate adenocarcinoma; READ, rectum adenocarcinoma; SARC, sarcoma; SKCM, skin cutaneous melanoma; STAD, stomach adenocarcinoma; STES, stomach and esophageal carcinoma; TGCT, testicular germ cell tumors; THCA, thyroid carcinoma; THYM, thymoma; UCEC, uterine corpus endometrial carcinoma; UCS, uterine carcinosarcoma; UVM, uveal melanoma

**FIGURE 2:**
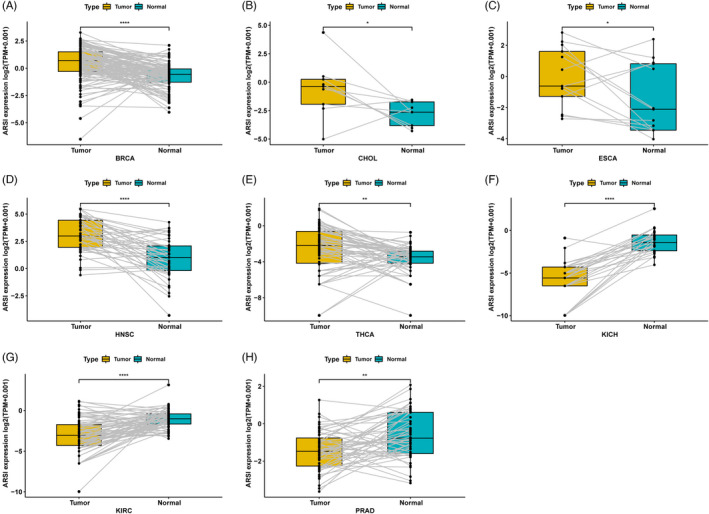
Comparison of *ARSI* gene expression between normal and tumor tissues. (A–E) High *ARSI* expression in BRCA, CHOL, ESCA, HNSC, and THCA. (F–H) Low *ARSI* expression in KICH, KIRC, and PRAD

Analyses of data from TCGA and GTEx databases revealed that *ARSI* expressions in BRCA, CHOL, DLBC, ESCA, GBM, HNSC, LGG, LIHC, OV, PAAD, SKCM, STAD, TGCT, THCA, and THYM tumors were higher, compared with their corresponding normal samples (*p* < 0.05; Figure 1A). In 33 tumor types, HNSC exhibited the highest levels of *ARSI*, followed by MESO (Figure 1B). Analysis of physiologic *ARSI* gene expressions across tissues using the GTEx data set (Figure 1C) revealed elevated expressions in lungs and lowest levels in blood. In addition, *ARSI* expressions were elevated in TCGA BRCA, CHOL, ESCA, HNSC, and THCA cohorts, compared with adjacent normal tissues (Figure 2A–E). Thus, *ARSI* may have a significant role in HNSC pathogenesis.

### 3.2 The landscape of *ARSI* genetic alterations in cancer

**FIGURE 3:**
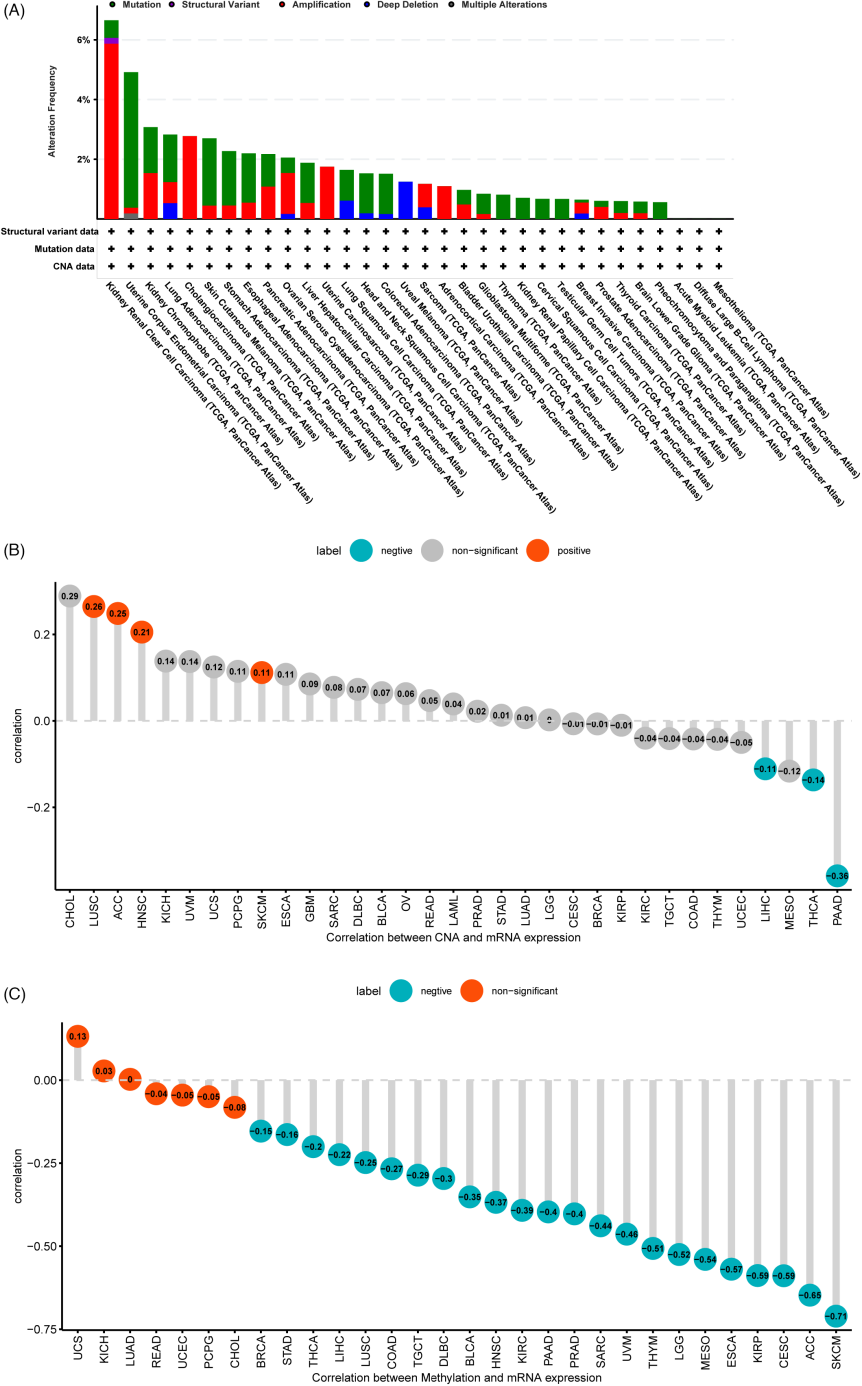
The genetic changes of *ARSI*. (A) *ARSI* mutation levels in cancers and ranked them from high to low based on cBioPortal database. (B) Correlation between CNA and *ARSI* mRNA expression. (C) Correlation between methylation and *ARSI* mRNA expression

cBioPortal, which has more than 28,000 tumor samples, was used to investigate genetic alterations of *ARSI*. It was revealed that KIRC had high mutation levels with an *ARSI* alteration frequency exceeding 6% (Figure 3A). The *ARSI* genetic alterations were mainly associated with HNSC. Furthermore, there were positive correlations between CNA and mRNA levels of *ARSI* in LUSC, ACC, HNSC, and SKCM, but negative correlations in PAAD, THCA, and LICH (Figure 3B). Methylation levels of the *ARSI* promoter were negatively correlated with *ARSI* expressions in 23 cancer types and were most pronounced in SKCM (Figure 3C).

### 3.3 Prognostic significance of *ARSI* in pan‐cancer

**FIGURE 4:**
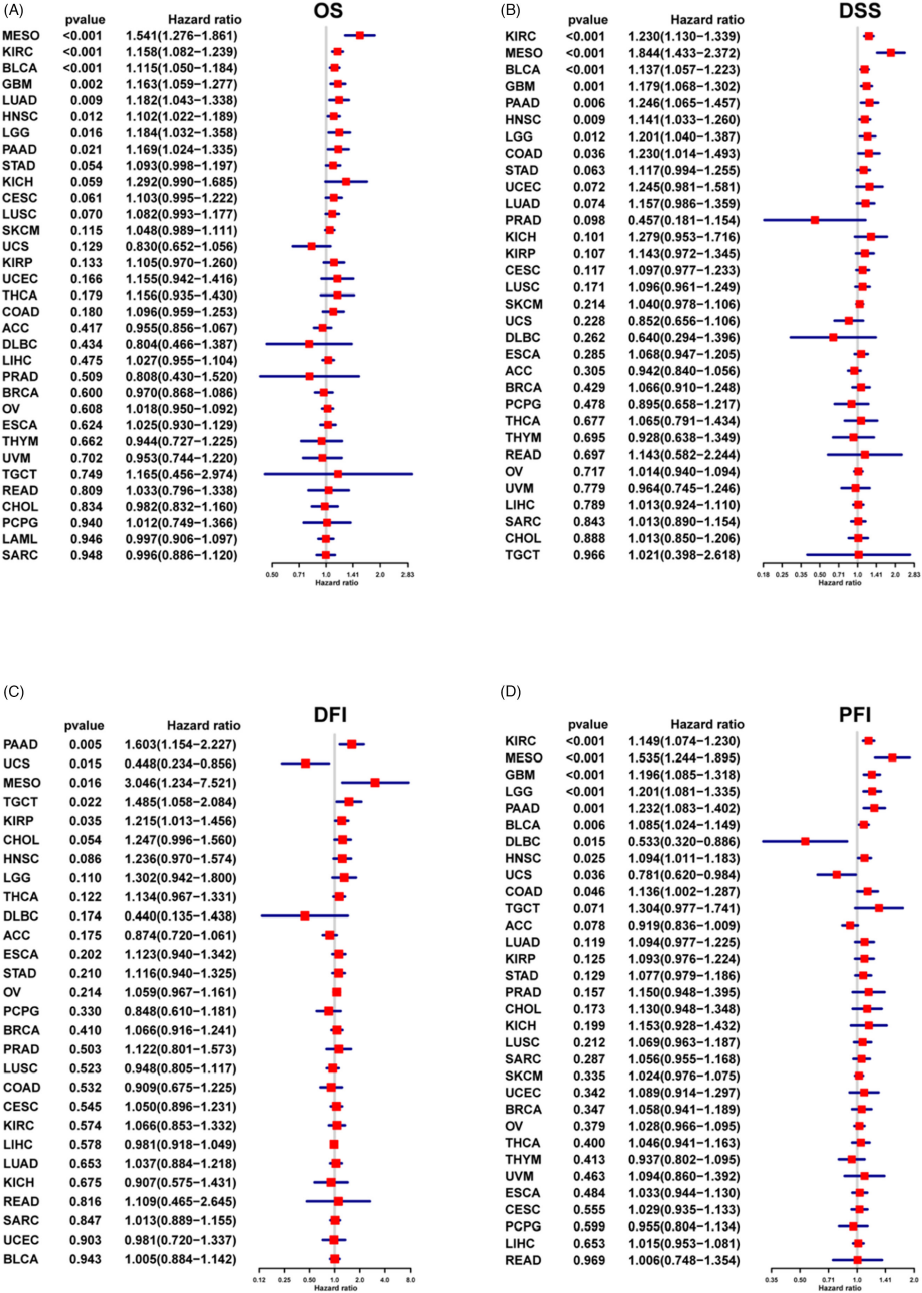
Prognostic value of *ARSI* in pan‐cancer. (A) The *ARSI* expression significantly correlated with OS in 8 types of cancer. (B) The *ARSI* expression significantly correlated with DSS in 8 types of cancer. (C) The *ARSI* expression significantly correlated with DFI in 5 types of cancer. (D) The *ARSI* expression significantly correlated with PFI in 10 types of cancer

**FIGURE 5:**
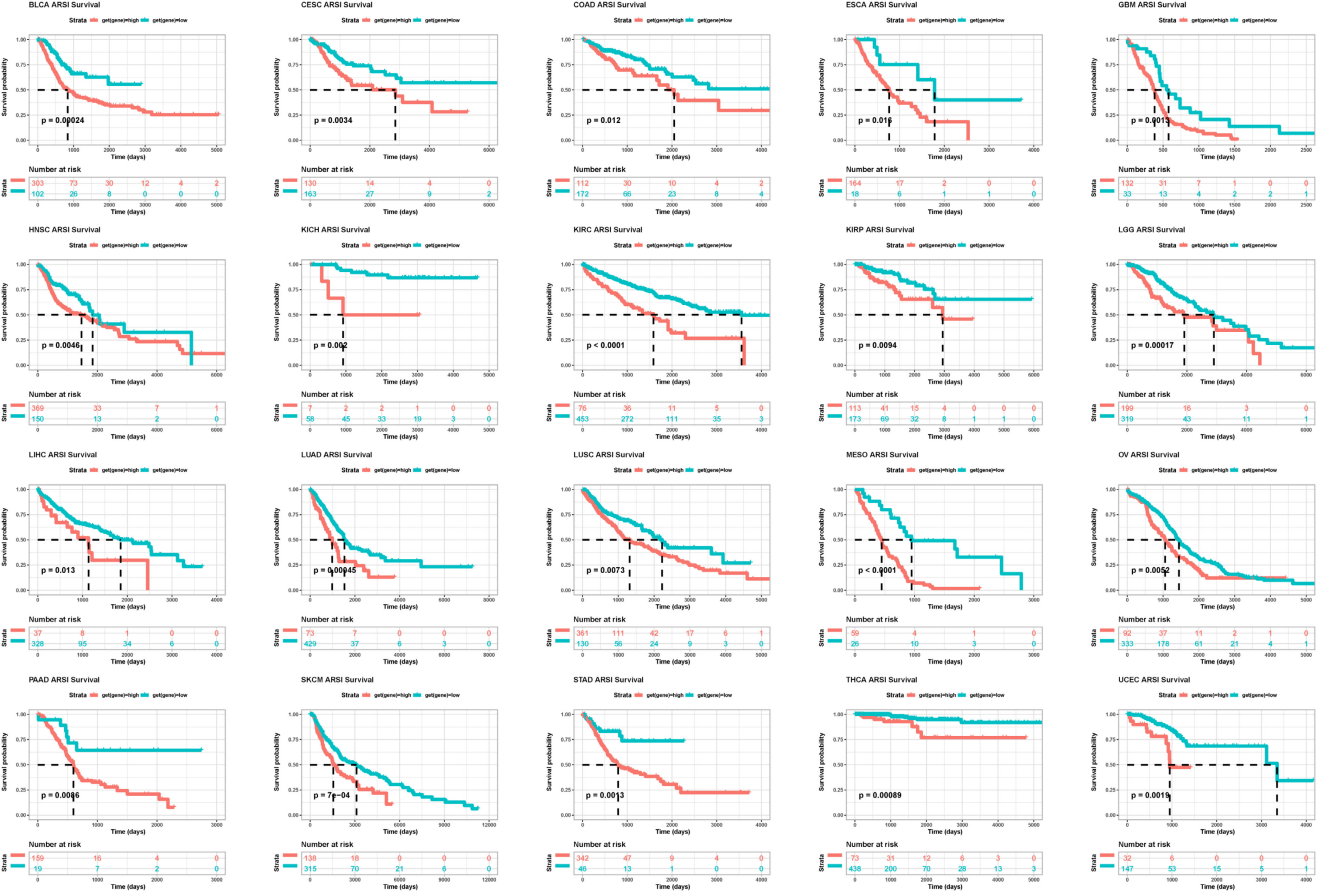
Kaplan–Meier analysis of the association between *ARSI* expression and OS in BLCA, CESC, COAD, ESCA, HNSC, KICH, KIRC, LIHC, LUAD, LUSC, MESO, SKCM, STAD, THCA, and UCEC

The overall survival analysis revealed that *ARSI* is a risk factor in patients with MESO, KIRC, BLCA, GBM, LUAD, HNSC, LGG, or PAAD, particularly in MESO (Figure 4A). The DSS analysis revealed significantly high hazard ratios for the *ARSI* gene in KIRC, MESO, BLCA, GBM, PAAD, HNSC, LGG, and COAD (Figure 4B). The DFI analysis showed that higher *ARSI* expressions were associated with poorer DFI in PAAD, MESO, TGCT, and KIRP. In contrast, elevated *ARSI* expressions were significantly associated with better DFI in UCS (Figure 4C). *ARSI* was found to be a protective factor for patients with DLBC and UCS, and a risk factor for patients with KIRC, MESO, GBM, LGG, PAAD, BLCA, HNSC, and COAD (Figure 4D). Kaplan–Meier survival analysis was used to study the association between *ARSI* expressions and prognostic outcomes in various cancers. *ARSI* was found to be a high‐risk gene in 20 cancer types, including BLCA, CESC, COAD, ESCA, HNSC, KICH, KIRC, LIHC, LUAD, LUSC, MESO, SKCM, STAD, THCA, and UCEC (Figure 5). The GSE41613 dataset was used for survival analysis to validate the survival value of *ARSI* (Figure S1).

### 3.4 GSVA of *ARSI*

**FIGURE 6:**
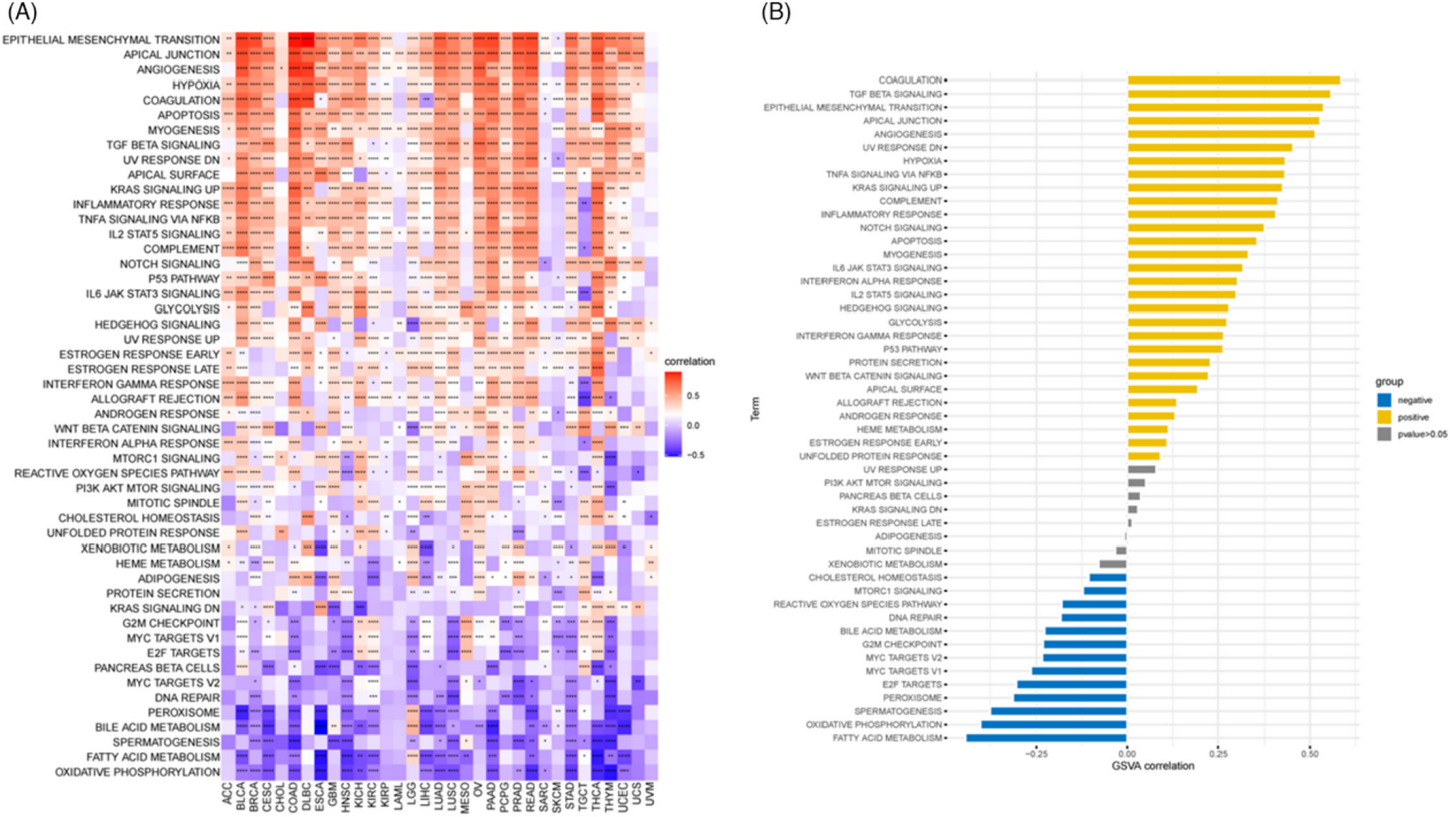
Results of GSVA. (A) The correlation of *ARSI* with 50 HALLMARK terms in pan cancer. (B) The results of GSVA analysis in HNSC. Yellow bars show the 29 pathways with the most significant positive correlation and blue bars show the 13 pathways with the most significant negative correlations

To assess the biological significance of *ARSI* expressions in different tumor tissues, GSVA was performed to compare gene expressions in 33 tumors with 50 gene sets. Figure 6A shows that some pathways were positively or negatively associated with *ARSI* expressions in various tumors. In most cancers, *ARSI* had significant positive correlations with 27 cancer‐related Hallmark pathways, including “EPITHELIAL MESENCHYMAL TRANSITION,” “APICAL JUNCTION,” “ANGIOGENECIS,” “HYPOXIA,” “COAGULATION,” “APOPTOSIS,” “MYOGENESIS,” “TGF BETA SIGNALING,” “UV RESPONSE DN,” “APICAI SURFACE,” “KRAS SIGNALING UP,” “INFLAMMATORY RESPONSE,” “TNFA SIGNALING VIA NFKB,” “IL2 STATS SIGNALING,” “COMPLEMENT,” “NOTCH SIGNALING,” “P53 PATHWAY,” “IL6 JAK STAT3 SIGNALING,” “GLYCOLYSIS,” “HEDGEHOG SIGNALING,” “UV RESPONSE UP,” “ESTROGEN RESPONSE EARIY,” “ESTROGEN RESPONSE LATE ” “INTERFERON GAMMA RESPONSE,” “ALLOGRAFT REJECTION,” “ANDROGEN RESPONSE,” and “WNIERPTACAI‐MINISIGNAHING.” In addition, *ARSI* exhibited positive correlations with other pathways such as “INTERFERON ALPHA RESPONSE,” “IL2 STAT5 SIGNALING,” “PROTEIN SECRETION,” “WNT BETA CATENIN SIGNALING,” “APICAL SURFACE,” “HEME METABOLISM,” and “UNFOLDED PROTEIN RESPONSE” in HNSC (Figure 6B).

### 3.5 GSEA enrichment analysis of *ARSI*

**FIGURE 7:**
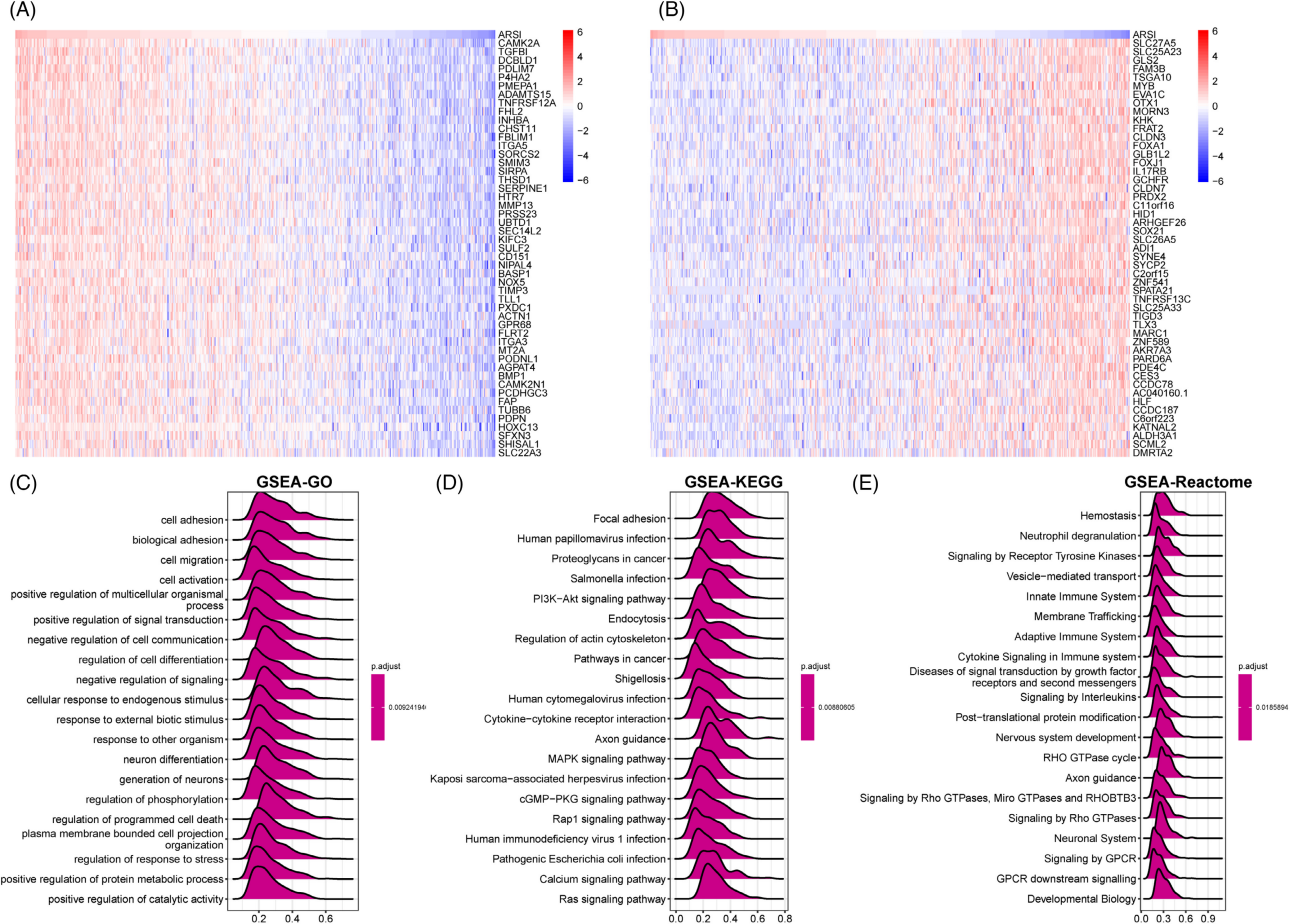
Functional Enrichment of GO and KEGG terms on *ARSI* through GSEA. (A) The heatmaps depicted the top 50 genes positively correlated to *ARSI*. (B) The heatmaps depicted the top 50 genes negatively correlated to ARSI. (C‐E) Merged plots of GSEA indicating the signaling pathways associated with *ARSI* expression according to GO, KEGG, and Reactome analyses in HNSC

The Spearman test was used to identify differentially expressed genes (DEGs) that were positively and negatively correlated with *ARSI* in HNSC. The top 50 positively (*r* > 0) and top 50 negatively (*r* < 0) correlated genes are shown in heat maps (Figure 7A,B). Thereafter, based on correlation analysis results, GSEA enrichment analysis was performed using “clusterpofiler” in R, which included GO, KEGG, and Reactome annotations. It was established that *ARSI* was significantly associated with pathways that mediate tumor cell invasion, migration, and metastasis in HNSC (Figure 7C–E).

### 3.6 Correlations between *ARSI* and tumor microenvironment (TME)

**FIGURE 8:**
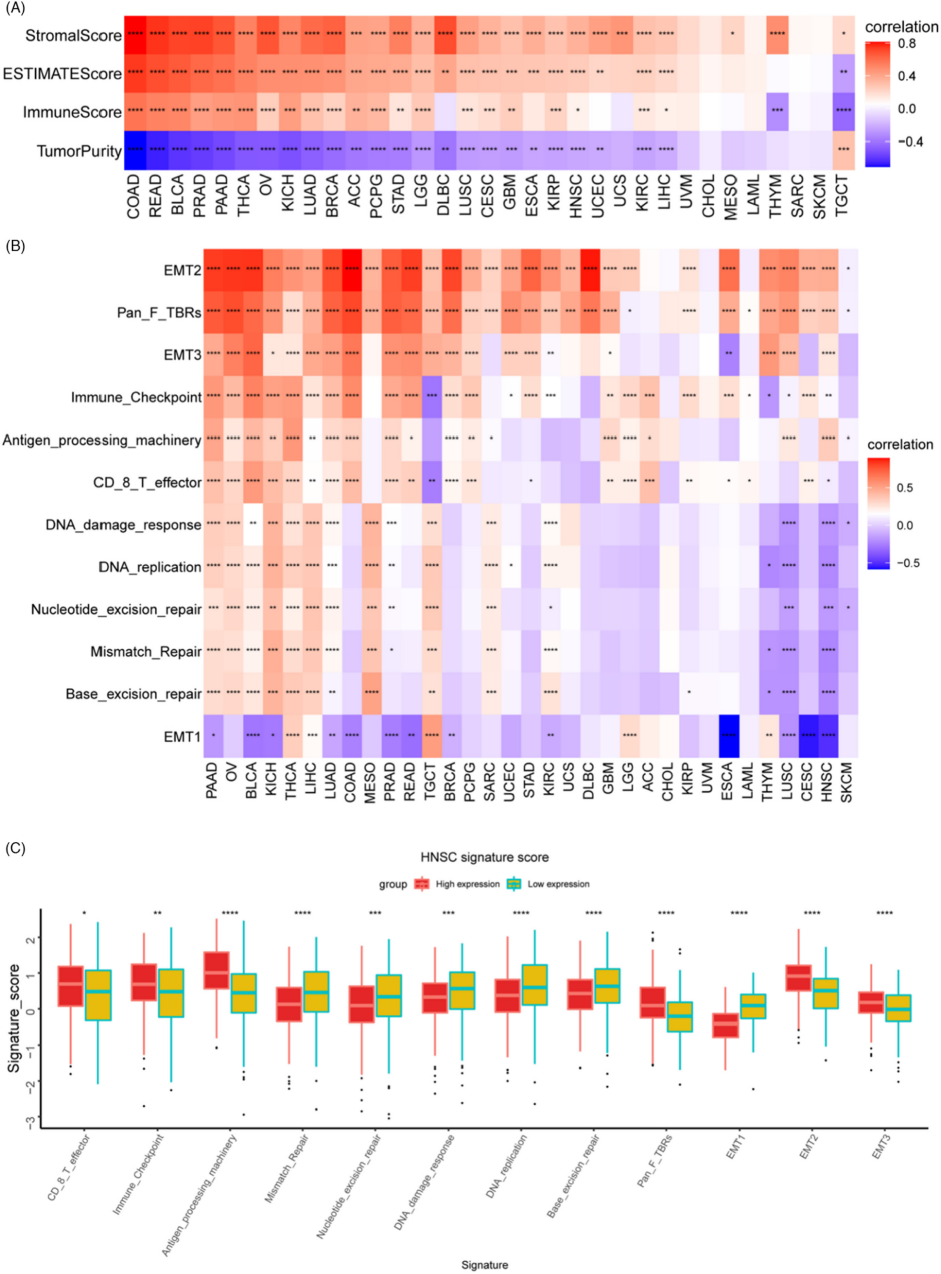
Correlation between *ARSI* and tumor microenvironment. (A) The correlation of *ARSI* with Immune Score, Stromal Score ESTIMATE score, and tumor purity. (B) The correlation of *ARSI* with immune‐related, DNA repair damage and metastasis‐related pathways in 33 cancers. (C) Boxplot depicted the correlation between *ARSI* transcript levels and signature score of pathways in HNSC

To determine whether *ARSI* is involved in immune cell infiltrations in the TME, the “ESTIMATE” package was used to evaluate the associations between *ARSI* expressions and stromal, immune, and ESTIMATE scores or tumor purity (Figure 8A). *ARSI* exhibited the highest correlations with stromal scores and immune scores in COAD. Besides, apart from TGCT, *ARSI* was negatively correlated with tumor purity in 24 cancer types (*p* < 0.05). The relationships between *ARSI* expressions and immune‐related genes, DNA repair damage, and metastasis‐related pathways were also assessed. There were significant positive correlations between elevated *ARSI* expressions and the above‐mentioned pathways in PAAD, apart from EMT1 (Figure 8B). Correlations between *ARSI* transcript levels and signature scores of pathways in HNSC are shown in a boxplot (Figure 8C).

**FIGURE 9:**
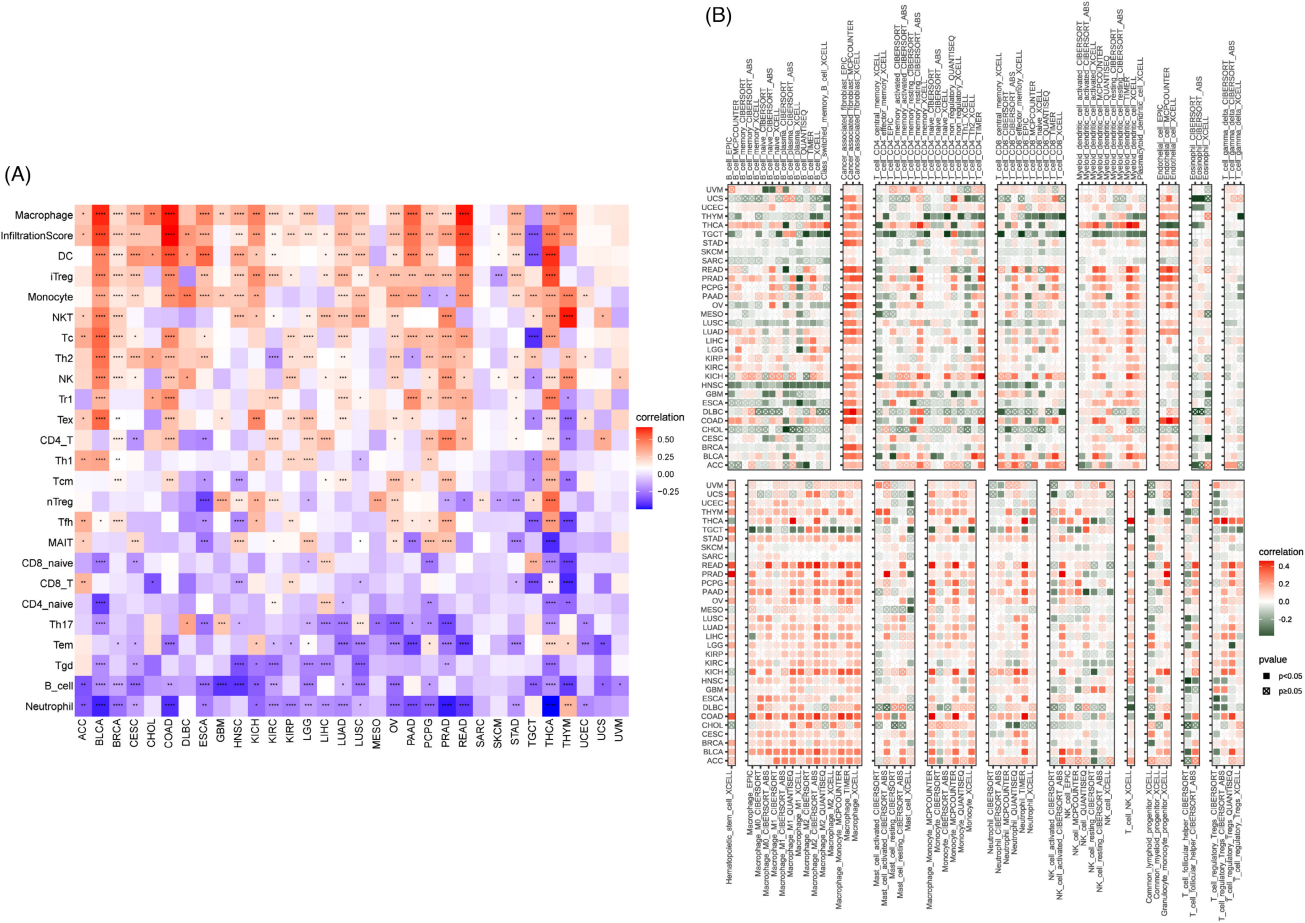
Correlation between *ARSI* expression and levels of tumor infiltration across different immune cells. (A) Correlation between ARSI expression and tumor infiltration of different immune cells from ImmuCellAI database. (B) Correlation between ARSI and different immune cells from TIMER database

Analysis of data from the ImmuCellAI database revealed that *ARSI* was positively correlated with infiltration levels of macrophages, DC, iTerg, monocytes, and NKT cells, but negatively correlated with neutrophils, B cells, Tgd, Tem, and Th17 cells in TCGA pan‐cancer (Figure 9A). Analysis of data from the TIMER database showed that *ARSI* was positively correlated with infiltration levels of macrophages, cancer‐associated fibroblasts, DC, endothelial cells, and monocytes, but negatively correlated with neutrophils, B cells, and follicular helper T cells in TCGA pan‐cancer (Figure 9B). The GSE41613 dataset was used to validate that *ARSI* is important for immune cell infiltrations in the HNSC microenvironment (Figure S2).

### 3.7 Associations between *ARSI* and tumor immune responses

**FIGURE 10:**
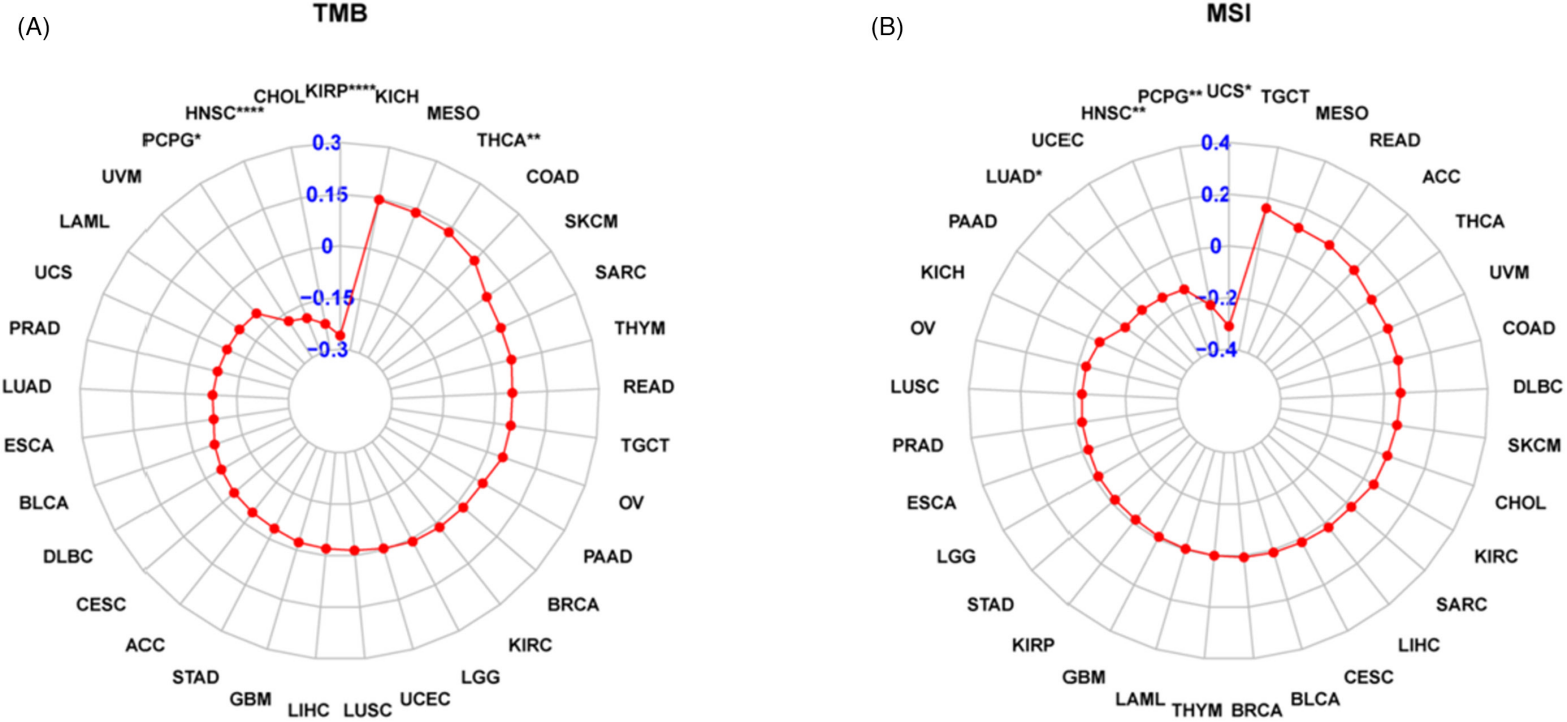
Correlation of *ARSI* with TMB (A) and MSI (B) in pan‐cancer analysis

**FIGURE 11:**
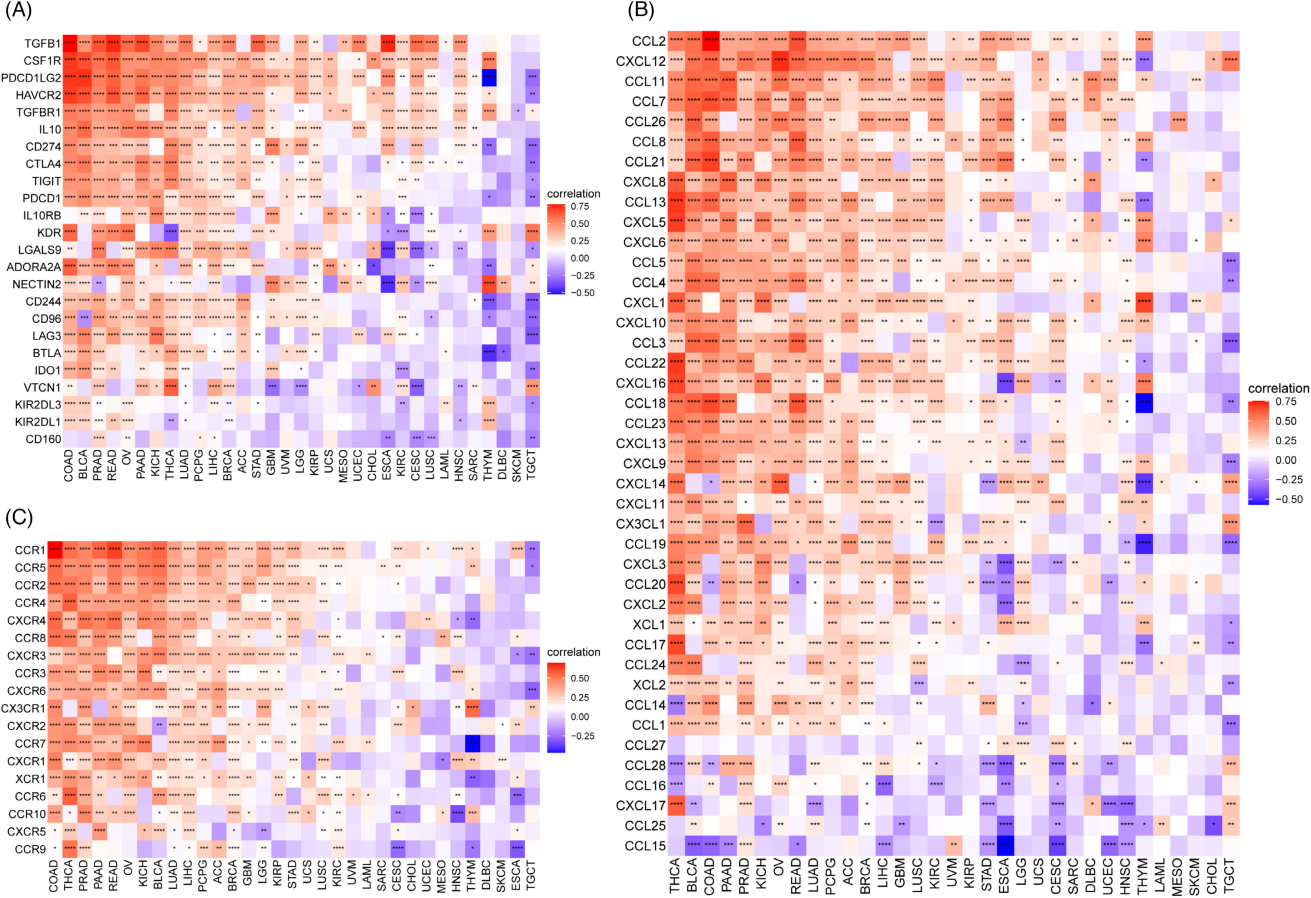
Co‐expression of *ARSI* and immune‐related genes. Immunosuppressive associated genes (A). Chemokine associated genes (B) Chemokine receptor associated genes (C). Red represents positive correlation, blue represents negative correlation

To define the roles of *ARSI* in immune mechanisms and responses, we assessed the interactions between *ARSI* levels and TMB, MSI, or IRGs, which could represent tumor immunogenicity and predict responses to immunotherapeutic agents. Figure 10A shows that *ARSI* mRNA expressions were negatively correlated with TMB in KIRP, HNSC, and PCPG, and positively correlated with THCA. Moreover, *ARSI* mRNA expressions exhibited negative correlations with MSI in UCS, PCPG, and HNSC (Figure 10B). Pan‐cancer analysis showed that *ARSI* mRNA expressions were associated with almost all immunosuppressive‐associated genes in most cancers, apart from DLBC and SKCM (Figure 11A–C). Among these cancer types, THCA, BLCA, COAD, PAAD, PRAD, KICH, OV, READ, LUAD, PCPG, ACC, and BRCA exhibited the highest coefficients, implying positive correlations between *ARSI* mRNA expressions and chemokine‐ or chemokine receptor‐associated genes.

### 3.8 *ARSI* and drug sensitivity

**FIGURE 12:**
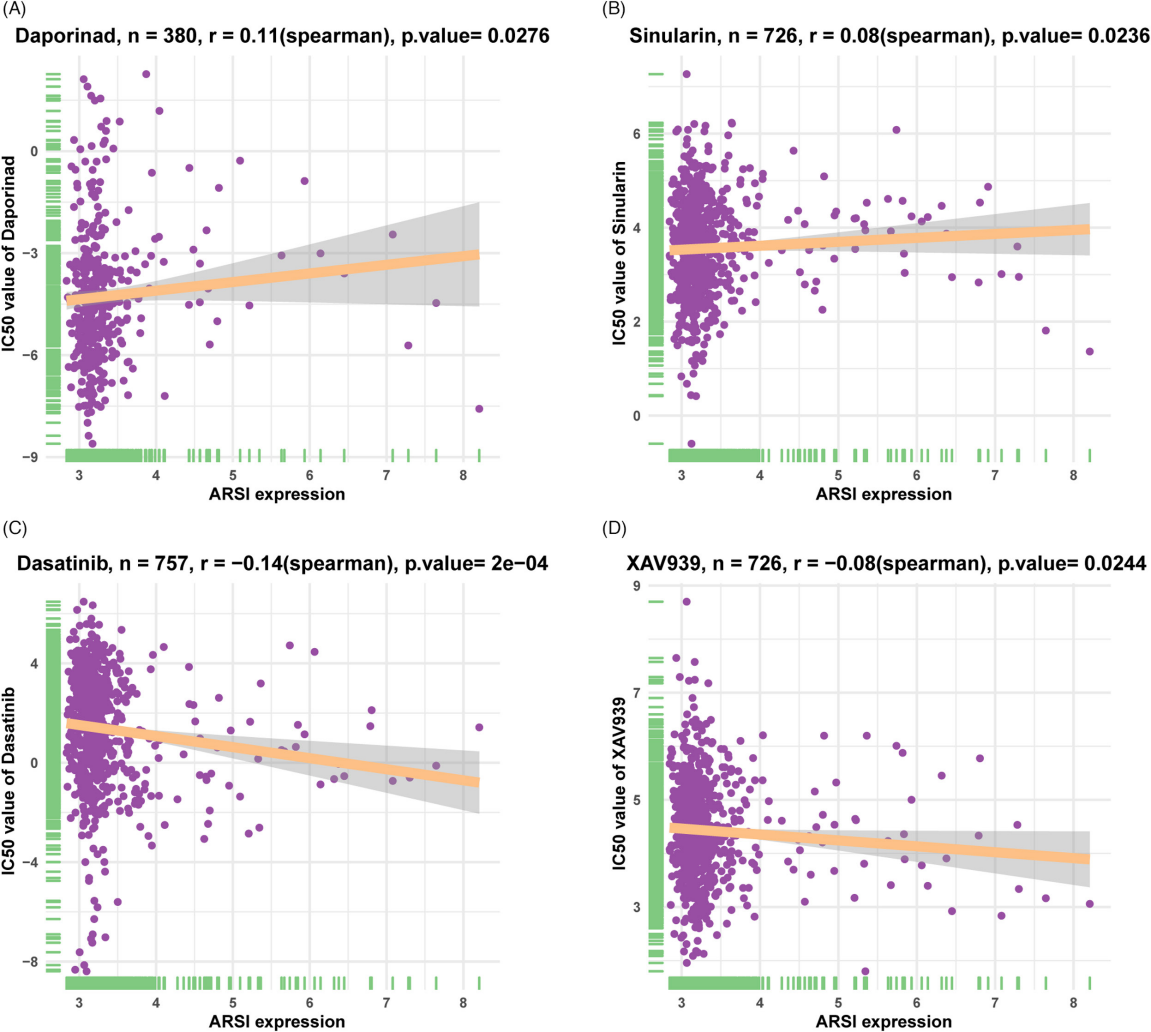
The correlation between the expression of *ARSI* and drug sensitivity. Higher *ARSI* expression had poor tolerance to daporinad (A) and sinularin (B) and were slightly sensitive to dasatinib (C) and XAV939 (D).

The GDSC data were used for drug sensitivity analysis of the *ARSI* gene via Spearman correlation tests, which yielded Spearman correlation coefficients. The top 2 positively (*r* > 0) and top 2 negatively (*r* < 0) correlated drugs were assessed using the “ggplot2” in R. Figure 12 shows that elevated expressions of *ARSI* conferred lower tolerance of cells to daporinad and sinularin, but increased cell sensitivity to dasatinib and XAV939.

## 4 DISCUSSION

A combination of molecular‐targeted therapies with immune checkpoint inhibitors (ICIs) is an effective therapeutic approach for cancer patients, especially HNSC.^10^, ^11^, ^12^, ^13^, ^14^ Although some of the molecular‐targeted drugs and ICIs for HNSC, such as anti‐PD‐1 antibodies, nivolumab, and pembrolizumab, are effective, genetic changes in patients alter therapeutic responses. Therefore, it is important to identify and validate efficient prognostic and diagnostic biomarkers in HNSC.

Arylsulfatase I is different from all the other members of sulfatase family that span across 8–20 exons.^15^ ARSI, a secreted protein that functions in the extracellular environment, is rapidly degraded in the endoplasmic reticulum (ER) or medium. Excess ARSI is retained in the ER, in a denatured form, to lead to SUMF1 degradation.^16^ Expressions of *ARSI* have been evaluated in different human tissues and cancer cell lines.^9^ *ARSI* was found to be principally expressed in embryonic tissues and in the A549 cell line, which originates from lung carcinoma. *ARSI* is involved in tissue remodeling during tumor growth, as well as during embryonic development. Even though the *ARSI* gene has been studied, its role in tumorigenesis has not been fully established.

In this study, we found that *ARSI* levels were highly elevated in tumor tissues, especially HNSC, compared with normal or adjacent non‐tumor tissues. In addition, there was a positive correlation between CNA and *ARSI* mRNA expressions and negative correlations with dysregulated methylation levels of the *ARSI* promoter in HNSC. These results suggest that overexpressions and genetic changes in *ARSI* mediate HNSC tumorigenesis. The prognostic value of *ARSI* has been reported in various cancer types, including HNSC. Our survival assays revealed that elevated *ARSI* expressions were associated with poorer prognostic outcomes (OS, DSS, and PFI), relative to low *ARSI* expressions. In contrast, although DFI was not significantly correlated with *ARSI* expressions (*p* = 0.086), there was a trend toward worse DFI for patients with high *ARSI* mRNA expressions (HR = 1.236, 95% CI = 0.970, 1.574) in univariate analysis. These findings imply that *ARSI* is a potential prognostic indicator in HNSC.

Our GSVA analysis showed that elevated *ARSI* expressions in HNSC were associated with consistent and significant dysregulation of coagulation, transforming growth factor (*TGF*)‐β signaling, epithelial–mesenchymal transition (EMT), apical junction, and angiogenesis gene sets. Furthermore, GSEA analysis showed that cell adhesion, biological adhesion, cell migration, or cell activation were potential mechanisms for HNSC progression. These results imply that *ARSI* may be associated with cell adhesion and cell migration in the TME, facilitating cancer cell migration and invasion.

In this study, *ARSI* expressions were associated with tumor‐infiltrating immune cells, which could influence tumor behaviors in multiple cancer types. For instance, *ARSI* was positively correlated with infiltrations of macrophages, monocytes, and cancer‐associated fibroblasts and negatively correlated with B cells, CD8+ T cells, and follicular helper T cells in HNSC. Macrophages and monocytes exhibited several protumorigenic abilities, which promoted tumor cell proliferation and metastasis.^17^, ^18^ Cancer‐associated fibroblasts secrete numerous extracellular matrix molecules, chemokines, cytokines, and growth factors to create a favorable microenvironment for tumor progression and invasion.^19^, ^20^ We found that *ARSI* was positively correlated with stromal, immune, and ESTIMATE scores in HNSC. Besides, *ARSI* expressions were significantly and positively associated with immune‐related and metastasis‐related pathways, but negatively correlated with DNA damage‐related and repair‐related pathways. Data suggest that *ARSI* may be involved in tumor immune evasion, leading to poor prognosis of HNSC.

Tumor mutation burden is a reliable biomarker for immunotherapeutic responses.^21^ Cancer patients with high TMB have poor prognostic outcomes. High‐MSI tumor may have a favorable inflammatory TME and a better sensitivity to ICIs.^22^, ^23^ Besides, IRGs play critical roles in transcriptional and microenvironmental alterations, and represent a novel predictor of clinical efficacy in cancer.^24^ Therefore, we assessed the relationship between *ARSI* expressions and TMB/MSI/IRGs. We found that *ARSI* expressions had strong negative correlations with TMB and MSI but slightly negative correlations with IRGs in HNSC. These findings show that TMB, MSI, and IRGs mediate the effects of *ARSI* in molecular‐targeted therapies and ICIs prognosis. Spearman correlation tests showed that HNSC patients with upregulated *ARSI* levels had poor tolerance to daporinad (*r* = 0.11) and sinularin (*r* = 0.08), but were slightly sensitive to dasatinib (*r* = −0.14) and XAV939 (*r* = −0.08). These results suggest that HNSC patients with high *ARSI* expressions may not be suitable for immunosuppressive therapy.

Although our study analyzed *ARSI* expressions in 33 tumors, our data sources were mainly derived from UCSC XENA and GDSC databases. Besides, we only used retrospective data; thus, there is a need for further validation in larger, prospective clinical trials. In addition, we found that *ARSI* expressions were associated with immune cell infiltrations and tumor metastasis, but not their causality.

In conclusion, *ARSI* is a promising prognostic biomarker in pan‐cancer, especially HNSC. These findings may inform clinical decisions and cancer treatment.

## FUNDING INFORMATION

This study was funded by Ningbo Health Branding Subject Fund (No.PPXK2018‐02); Zhejiang Provincial Natural Science Foundation of China (LY19H160014; LQ21H130001); Ningbo “Technology Innovation 2025” Major Special Project (2020Z097; 2018B10015); Medical and Health Research Project of Zhejiang Province (2019ZD018; 2021KY307).

## CONFLICT OF INTEREST

The authors declare that they have no competing interests.

## Supporting information


Figure S1‐S2

## Data Availability

The data that support the findings of this study are openly available in UCSC XENA website (https://xenabrowser.net/datapages/ and DOI: 10.1038/s41587‐020‐0546‐8), the Gene Expression Omnibus (GEO) database, cBioPortal (https://www.cbioportal.org/ and DOI: 10.1136/gut.2007.143065), TIMER (http://timer.cistrome.org and DOI: 10.1093/nar/gkaa407), and GDSC (https://www.cancerrxgene.org and DOI: 10.1093/nar/gks1111).

## References

[1] Kang S , Elf S , Lythgoe K , et al. p90 ribosomal S6 kinase 2 promotes invasion and metastasis of human head and neck squamous cell carcinoma cells. J Clin Invest. 2010;120(4):1165‐1177. 20234090 10.1172/JCI40582 PMC2846050

[2] Chung CH , Zhang Q , Kong CS , et al. p16 protein expression and human papillomavirus status as prognostic biomarkers of nonoropharyngeal head and neck squamous cell carcinoma. J Clin Oncol. 2014;32(35):3930‐3938. 25267748 10.1200/JCO.2013.54.5228 PMC4251957

[3] van Luijk P , Pringle S , Deasy JO , et al. Sparing the region of the salivary gland containing stem cells preserves saliva production after radiotherapy for head and neck cancer. Sci Transl Med. 2015;7(305):305ra147. 10.1126/scitranslmed.aac4441 PMC4964284 26378247

[4] Zhao H , Wu L , Yan G , et al. Inflammation and tumor progression: signaling pathways and targeted intervention. Signal Transduct Target Ther. 2021;6(1):263. 34248142 10.1038/s41392-021-00658-5 PMC8273155

[5] Faubert B , Solmonson A , DeBerardinis RJ . Metabolic reprogramming and cancer progression. Science. 2020;368(6487):eaaw5473. 32273439 10.1126/science.aaw5473 PMC7227780

[6] Hanahan D . Hallmarks of cancer: new dimensions. Cancer Discov. 2022;12(1):31‐46. 35022204 10.1158/2159-8290.CD-21-1059

[7] Lübke T , Damme M . Lysosomal sulfatases: a growing family. Biochem J. 2020;477(20):3963‐3983. 33120425 10.1042/BCJ20200586

[8] Habuchi H , Habuchi O , Kimata K . Sulfation pattern in glycosaminoglycan: does it have a code? Glycoconj J. 2004;21(1–2):47‐52. 15467398 10.1023/B:GLYC.0000043747.87325.5e

[9] Obaya AJ . Molecular cloning and initial characterization of three novel human sulfatases. Gene. 2006;372:110‐117. 16500042 10.1016/j.gene.2005.12.023

[10] Qian Y , Gong Y , Fan Z , et al. Molecular alterations and targeted therapy in pancreatic ductal adenocarcinoma. J Hematol Oncol. 2020;13(1):130. 33008426 10.1186/s13045-020-00958-3 PMC7532113

[11] Nagano T , Tachihara M , Nishimura Y . Molecular mechanisms and targeted therapies including immunotherapy for non‐small cell lung cancer. Curr Cancer Drug Targets. 2019;19(8):595‐630. 30526458 10.2174/1568009619666181210114559

[12] Shin MH , Kim J , Lim SA , Kim J , Lee KM . Current insights into combination therapies with MAPK inhibitors and immune checkpoint blockade. Int J Mol Sci. 2020;21(7):2531. 10.3390/ijms21072531 PMC7177307 32260561

[13] Kelley RK , Bridgewater J , Gores GJ , Zhu AX . Systemic therapies for intrahepatic cholangiocarcinoma. J Hepatol. 2020;72(2):353‐363. 31954497 10.1016/j.jhep.2019.10.009

[14] Kitamura N , Sento S , Yoshizawa Y , Sasabe E , Kudo Y , Yamamoto T . Current trends and future prospects of molecular targeted therapy in head and neck squamous cell carcinoma. Int J Mol Sci. 2020;22(1):240. 10.3390/ijms22010240 PMC7795499 33383632

[15] Sardiello M , Annunziata I , Roma G , Ballabio A . Sulfatases and sulfatase modifying factors: an exclusive and promiscuous relationship. Hum Mol Genet. 2005;14(21):3203‐3217. 16174644 10.1093/hmg/ddi351

[16] Oshikawa M , Usami R , Kato S . Characterization of the arylsulfatase I (ARSI) gene preferentially expressed in the human retinal pigment epithelium cell line ARPE‐19. Mol Vis. 2009;15:482‐494. 19262745 PMC2650720

[17] Nielsen SR , Schmid MC . Macrophages as key drivers of cancer progression and metastasis. Mediators Inflamm. 2017;2017:9624760. 28210073 10.1155/2017/9624760 PMC5292164

[18] Hanna RN , Cekic C , Sag D , et al. Patrolling monocytes control tumor metastasis to the lung. Science. 2015;350(6263):985‐990. 26494174 10.1126/science.aac9407 PMC4869713

[19] Kwa MQ , Herum KM , Brakebusch C . Cancer‐associated fibroblasts: how do they contribute to metastasis? Clin Exp Metastasis. 2019;36(2):71‐86. 30847799 10.1007/s10585-019-09959-0

[20] Kubo N , Araki K , Kuwano H , Shirabe K . Cancer‐associated fibroblasts in hepatocellular carcinoma. World J Gastroenterol. 2016;22(30):6841‐6850. 27570421 10.3748/wjg.v22.i30.6841 PMC4974583

[21] Chan TA , Yarchoan M , Jaffee E , et al. Development of tumor mutation burden as an immunotherapy biomarker: utility for the oncology clinic. Ann Oncol. 2019;30(1):44‐56. 30395155 10.1093/annonc/mdy495 PMC6336005

[22] Diao Z , Han Y , Chen Y , Zhang R , Li J . The clinical utility of microsatellite instability in colorectal cancer. Crit Rev Oncol Hematol. 2021;157:103171. 33290824 10.1016/j.critrevonc.2020.103171

[23] van Velzen MJM , Derks S , van Grieken NCT , Haj Mohammad N , van Laarhoven HWM . MSI as a predictive factor for treatment outcome of gastroesophageal adenocarcinoma. Cancer Treat Rev. 2020;86:102024. 32388292 10.1016/j.ctrv.2020.102024

[24] Gnjatic S , Bronte V , Brunet LR , et al. Identifying baseline immune‐related biomarkers to predict clinical outcome of immunotherapy. J Immunother Cancer. 2017;5:44. 28515944 10.1186/s40425-017-0243-4 PMC5432988

