# Supplementary material for: Arylsulfatase I is a prognostic biomarker for head and neck squamous cell carcinoma and Pan‐cancer
Source: J Clin Lab Anal. 2022 Jul 23;36(9):e24600. doi: 10.1002/jcla.24600 (PMC9459304; doi:10.1002/jcla.24600)
Supplement: Supplementary file 1 — Figure S1‐S2 [file JCLA-36-e24600-s001.docx]

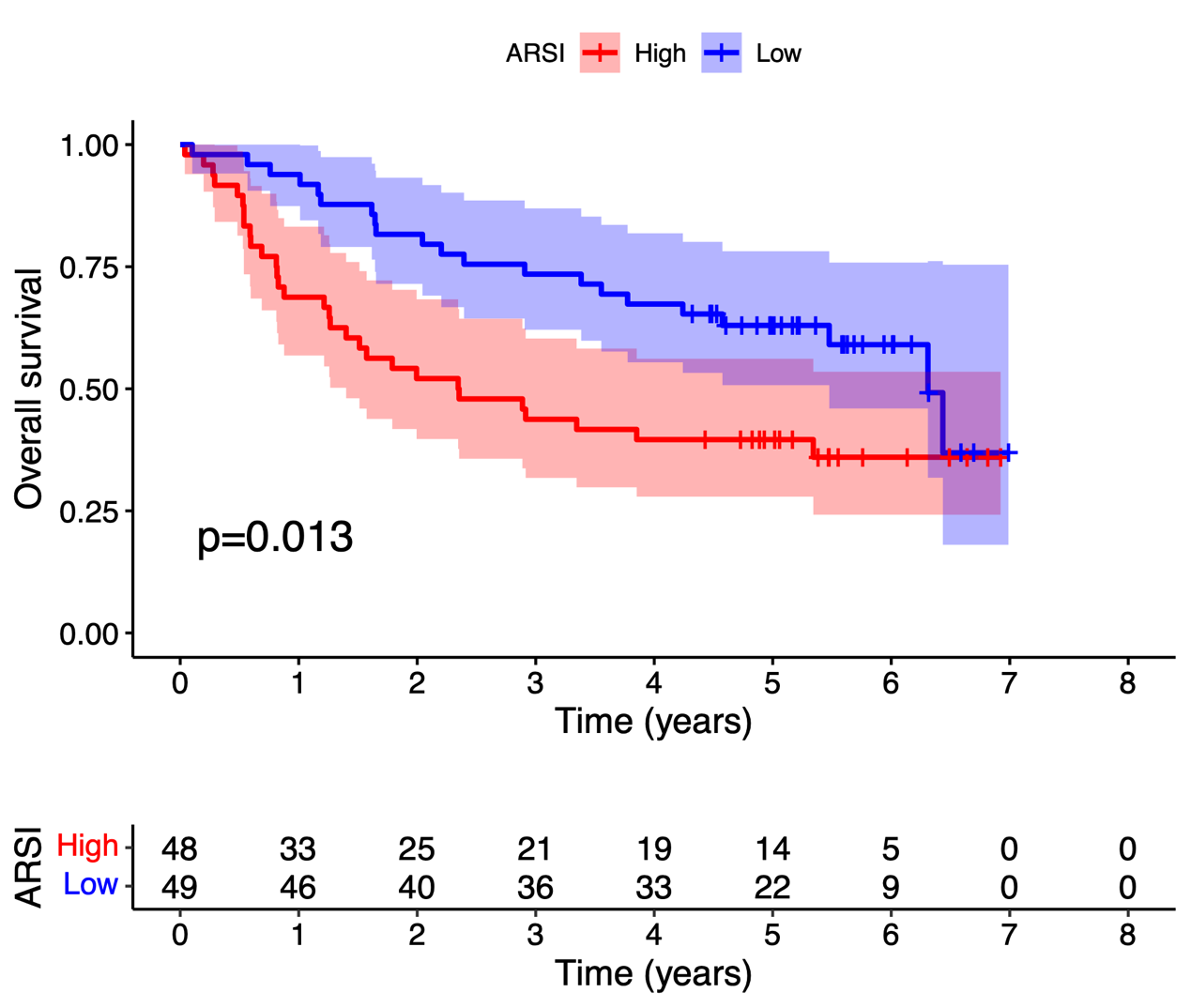


Figure S1: The survival value of *ARSI* in GSE41613.


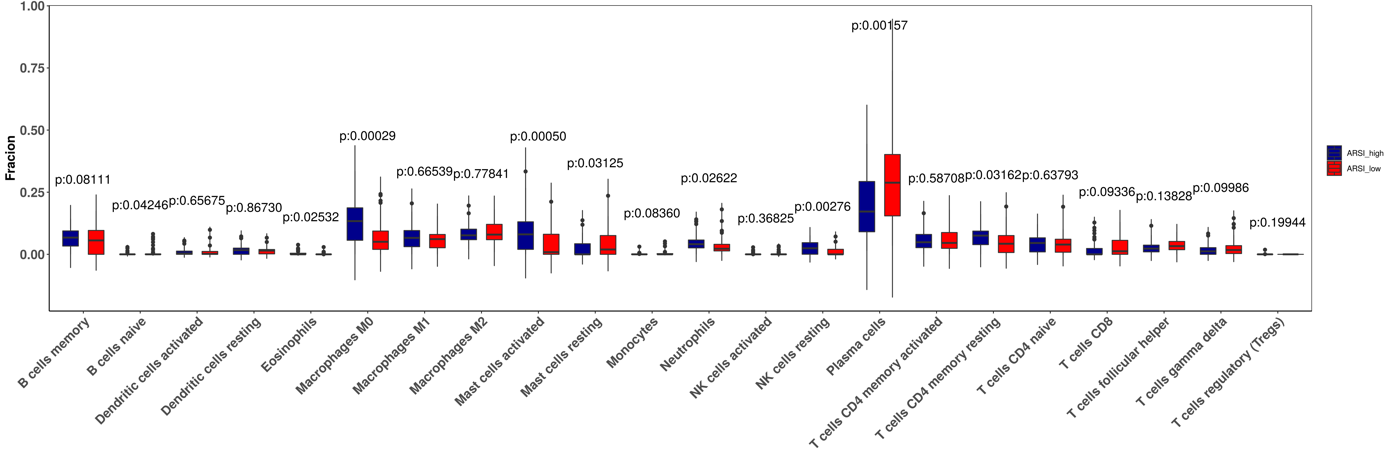


Figure S2: The relationship between *ARSI* expression and the infiltrating immune cells in GSE41613.
